# Tumeur de Brenner maligne avec très bonne réponse après chimiothérapie: à propos d'un cas et revue de la littérature

**DOI:** 10.11604/pamj.2014.17.293.2443

**Published:** 2014-04-17

**Authors:** Meryam Ben Ameur El Youbi, Hind M'rabti, Amina Mohtaram, Imane Aaribi, Jinane Kharmoum, Basma El Khannoussi, Hassan Errihani

**Affiliations:** 1 Service d'Oncologie Médicale, Institut National d'Oncologie, Rabat, Maroc; 2 Service d'Anatomie Pathologique, Institut National d'Oncologie, Rabat, Maroc

**Keywords:** Tumeur de Brenner maligne, ovaire, chirurgie, chimiothérapie, malignant Brenner tumor, ovary, surgery, chimiotherapy

## Abstract

La tumeur de Brenner est une lésion ovarienne rare représentant 1 à 2% de l'ensemble des tumeurs ovariennes. Elle a été décrite pour la première fois par MacNaughton-Jones en 1898. La tumeur de Brenner maligne est une variante exceptionnelle survenant dans 2 à 5% des cas de tumeurs de Brenner. Nous proposons de discuter à partir d'une observation clinique de tumeur de Brenner maligne diagnostiquée chez une patiente de 47 ans traitée par chimiothérapie puis chirurgie radicale, et à travers une revue de la littérature; les différentes particularités cliniques et anatomo-pathologiques de cette entité rare ainsi que leurs implications diagnostiques et thérapeutiques. Le traitement repose principalement sur la chirurgie. L'indication d'une chimiothérapie adjuvante ou néo-adjuvante reste controversée.

## Introduction

Les tumeurs de Brenner correspondent à des tumeurs ovariennes à cellules transitionnelles composées de cellules matures semblables aux cellules urothéliales formant des nids au sein d'un stroma fibromateux. Ces tumeurs peuvent être bénignes, borderline ou malignes. Les tumeurs de Brenner malignes posent un véritable problème de prise en charge thérapeutique de par leur extrême rareté et de leur pronostic réputé être mauvais. Dans cette observation, nous rapportons le cas d'une tumeur de Brenner maligne diagnostiquée chez une patiente de 47 ans, ayant présenté une très bonne réponse après chimiothérapie, avec une revue de la littérature des différents aspects clinques, histologiques et thérapeutiques de cette lésion ovarienne très rare.

## Patient et observation

Une patiente âgée de 47 ans, nulligeste et sans antécédents pathologiques notables s'est présentée à la consultation de gynécologie pour une distension abdominale progressive associée à des douleurs pelviennes et un trouble du cycle menstruel évoluant depuis 10 mois.

Une échographie abdomino-pelvienne avait révélé une volumineuse masse pelvienne gauche. Le taux de CA 125 était normal à 13.05 UI/L. A l'exploration chirurgicale, la cavité pelvienne était occupée par une énorme tumeur ovarienne gauche mesurant 24 cm de grand axe associée à une carcinose péritonéale diffuse du grand épiploon, du sigmoïde et du pelvis, non résécable d'emblée en totalité.

**Figure 1:**
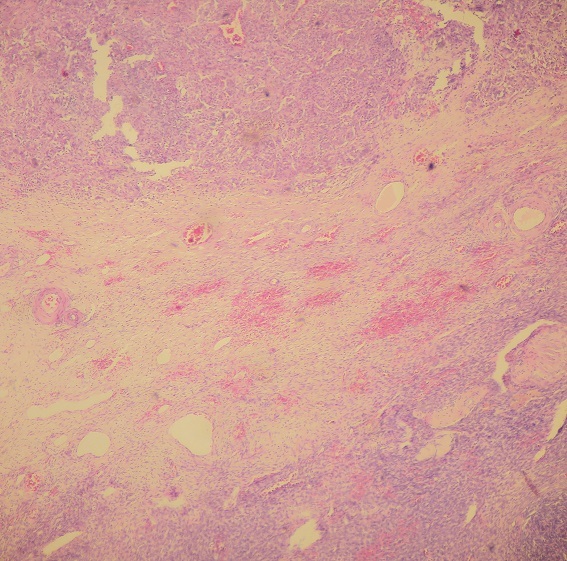
Aspect microscopique au faible grossissement (HE x 5) montrant la prolifération tumorale carcinomateuse et le stroma ovarien

**Figure 2:**
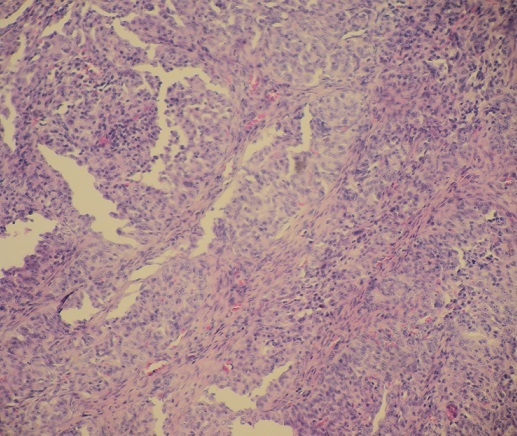
La prolifération tumorale est faite de papilles coalescentes à axe conjonctivo-vasculaire, de massifs et de petits amas (HE x 10)

**Figure 3:**
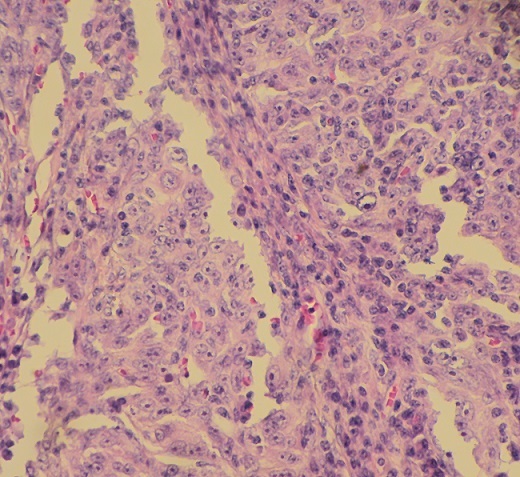
Les cellules tumorales présentent des atypies cytonucléaires (HE x 40)

Après annexectomie gauche et résection d'un nodule de carcinose; l'examen anatomo-pathologique avait conclu à une tumeur de Brenner maligne classée stade IIIc (Figure 1, Figure 2, Figure 3). Un scanner abdomino-pelvien réalisé en post-opératoire avait objectivé un aspect de carcinose péritonéale associée à des adénopathies lombo-aortiques gauches.

La stratégie thérapeutique proposée était celle d'une chimiothérapie première suivie d'une chirurgie d'intervalle en cas de bonne réponse. La patiente avait reçu par la suite une chimiothérapie intraveineuse à base de paclitaxel à la dose de 175 mg/m2 et carboplatine AUC 5 tous les 21 jours, avec une réponse clinique et radiologique complète après 3 cycles et bonne tolérance clinique et biologique. Le taux du CA 125 était resté normal à 10 UI/L. Puis elle avait bénéficié d'une chirurgie radicale associant une hystérectomie totale, une annexectomie droite et une omentectomie sous-colique.

**Figure 4:**
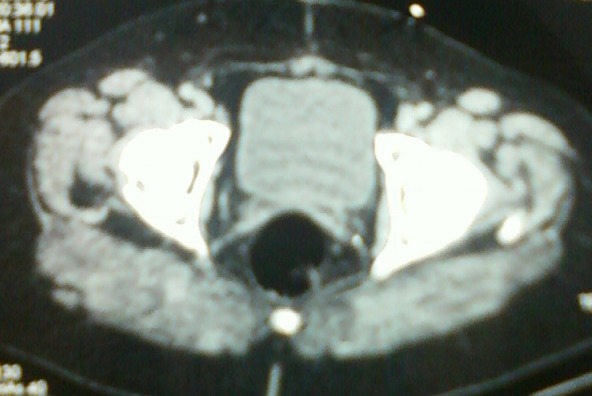
Vacuité de la loge génitale sans individualisation de masse en regard

L'examen histologique de la pièce opératoire avait objectivé une infiltration ovarienne droite et péritonéale massive par une prolifération carcinomateuse à cellules transitionnelles compatible avec une tumeur de Brenner maligne. Une surveillance a été préconisée. La patiente est toujours en bon contrôle clinique et radiologique (Figure 4).

## Discussion

La tumeur de Brenner a été décrite pour la première fois par MacNaughton-Jones en 1889 [1], puis par Brenner en 1907 qui l'avait comparé au follicule de De Graaf en raison de la forte ressemblance avec les nids épithéliaux, suspectant ainsi une origine à partir des cellules de la granulosa [2]. En 1932, Meyer avait permis de différencier les tumeurs des cellules de la granulosa de ce type de tumeur qu'il avait baptisé «tumeur de Brenner» [3]. Son caractère malin a été rapporté pour la première fois après plusieurs années dans une observation de Von Numers en 1942 [4].

Les tumeurs ovariennes constituées de cellules de type transitionnel ou excréto-urinaire sont désignées dans la classification de l'OMS de 2003 par le groupe des tumeurs à cellules transitionnelles. Cette classe de tumeurs comporte la tumeur de Brenner pouvant être bénigne, borderline ou maligne et le carcinome à cellules transitionnelles de type non Brenner.

Les tumeurs de Brenner sont une entité rare représentant 1 à 2% de l'ensemble des tumeurs ovariennes. Elles sont dans la majorité des cas bénignes. Cependant, seules 2 à 5% des tumeurs de Brenner sont malignes; posant ainsi des problèmes de diagnostic et de prise en charge thérapeutique [3].

Ces tumeurs surviennent chez la femme ménopausée âgée entre 50 et 70 ans. Les signes cliniques sont peu spécifiques, les douleurs pelviennes sont généralement au premier plan associées ou non à une masse pelvienne, un saignement vaginal anormal ou encore une irrégularité du cycle menstruel peuvent également être observés [5].

Sur le plan macroscopique, elles sont le plus souvent unilatérales, volumineuses mesurant 20 cm de diamètre et d'aspect blanc-grisâtre. Le caractère bilatéral est peu fréquent pouvant survenir dans 12% des cas. Elles se caractérisent généralement par la présence d'une composante solide correspondant à la tumeur de Brenner bénigne associée à des kystes contenant des masses papillaires ou polypoïdes.

Histologiquement, La tumeur de Brenner maligne est caractérisée par la présence de foyers typiques de carcinome à cellules transitionnelles de haut grade, rarement de bas grade, associés à une composante de tumeur de Brenner bénigne ou borderline baignant dans un stroma fibromateux [6]. Des éléments glandulaires ou des foyers de calcifications proéminents peuvent également y être associés [7]. La réalisation d'un échantillonnage étendu de la tumeur est nécessaire pour établir le diagnostic histologique exact [5]. Le diagnostic différentiel se pose essentiellement avec un carcinome séreux de haut grade. Sur la plan génétique, cette tumeur rejoint les carcinomes épithéliaux de type I avec des mutations portant essentiellement sur les gènes KRAS, BRAF et PTEN [8].

Le CA 125 qui est un marqueur sérique important dans l’évaluation des carcinomes ovariens; contribue peu dans le diagnostic ce type de tumeur, puisqu'il n'est pas spécifique [5]. Cependant un taux élevé du CA 125 peut prédire de leur nature maligne [9]. Des métastases ont été décrites dans 50% des cas et sont le plus souvent de localisations locorégionales. L'atteinte péritonéale étant la plus fréquente.

La prise en charge thérapeutique est calquée sur celle des autres cancers épithéliaux de l'ovaire et repose essentiellement sur la chirurgie de cytoréduction, consistant en une hystérectomie totale avec annexectomie bilatérale et omentectomie [3].

Concernant le traitement médical, le recours à une chimiothérapie adjuvante et encore moins néo-adjuvante est controversée selon les auteurs, en raison des faibles taux de réponses objectives et histologiques rapportés dans la littérature. Les réponses complètes histologiques sont exceptionnelles et ont été observées essentiellement après une polychimiothérapie à base de sels de platine [8]. Un schéma classique de type paclitaxel - carboplatine serait fortement recommandé [10].

En 1992, Platini avait rapporté deux cas de rémission histologique complète après polychimiothérapie chez deux patientes opérées pour tumeur de Brenner maligne classée stade IIIc; la première patiente avait reçu 6 cures de chimiothérapie associant cyclophosphamide et cisplatine, et la deuxième 6 cures à base de cyclophosphamide, doxorubicine et carboplatine. Dans les deux cas, la rémission histologique complète a été vérifiée par laparotomie de révision [9].

Dans une série publiée récemment par Gezginç et al, décrivant 13 cas de tumeur de Brenner maligne, toutes les patientes avaient bénéficié d'une chirurgie radicale d'emblée; une chimiothérapie post-opératoire à base de paclitaxel et carboplatine a été administrée chez 10 patientes; 9 d'entre elles étaient en réponse complète. Cependant, la majorité des patientes avaient reçu la chimiothérapie dans le cadre du traitement adjuvant, rendant le terme de réponse complète inapproprié dans ce cas là. Dans la même étude, la moitié des patientes avaient rechuté [10].

Chez notre patiente, l'administration de 3 cures de chimiothérapie à base de paclitaxel et carboplatine a permis d'obtenir une réponse radiologique et clinique complète, rendant la résécabilité tumorale possible et complète. Cependant, la réponse histologique n’était pas importante justifiant la surveillance post-opératoire.

La survie des stades Ia de la FIGO est estimée à 88% à 5 ans. Cependant, les tumeurs de Brenner malignes de stades avancés sont de mauvais pronostic avec une survie moyenne à 5 ans ne dépassant pas 40% [3].

## Conclusion

La tumeur de Brenner maligne est un diagnostic exceptionnel d'une tumeur ovarienne dont le pronostic reste réservé. En absence de standard thérapeutique, la prise en charge est essentiellement chirurgicale. L'indication d'une chimiothérapie reste discutable. Dans notre cas, la patiente est toujours en bon contrôle après un traitement optimal associant une chimiothérapie première à base de sels de platine et une chirurgie radicale.
